# Normative data for the letter-cancellation task in school children

**DOI:** 10.4103/0973-6131.43544

**Published:** 2008

**Authors:** Balaram Pradhan, H R Nagendra

**Affiliations:** Division of Yoga & Life Sciences, Swami Vivekananda Yoga Anusandhana Samsthana, Bangalore, India

**Keywords:** Attention, cancellation, information processing speed, psychomotor task, sustained

## Abstract

**Aims:**

To establish the norms for the letter-cancellation task—a psychomotor performance task.

**Materials and Methods:**

Eight hundred nineteen school students were selected in the present study in an age range between nine and 16 years (*M* = 12.14; *SD* = 1.78 years). Subjects were assessed once for the cancellation task.

**Results:**

Both age and sex influenced performance on the SLCT; therefore, correction scores were obtained on the basis of these factors.

**Conclusions:**

The availability of Indian normative data for the SLCT will allow wider application of this test in clinical practice.

## INTRODUCTION

Cancellation tests have a long history in neuropsychological assessment. Most commonly, they are administered as paper-and-pencil tests that are normally used to assess a person's ability to visually search for an identifiable target and to either cancel or circle all such target items in an array. They vary widely in their complexity from long letter strings, such as the "H" Test[1] and "A" Test[2] or number strings like the "2 and 7 Cancellation" Test.[3] They may include symbols that are quite simple as in the "Star Cancellation,"[4] Teddy Bear Cancellation,[5] and Symbol Cancellation tests.[6]

However, they have also been utilized in neuropsychological test batteries for the assessment of the effectiveness of treatment for adult patients with anorexia nervosa and bulimia nervosa,[7] and for the assessment of illiterate individuals to determine if education affected performance in a neuropsychological battery.[8] They have also been employed to assess cognitive impairments in alcoholic cirrhotic patients,[9] and to evaluate target detection deficits in patients who have undergone frontal lobectomy surgery.[10]

An individual's performance on cancellation tests often depends on their vigilance, motivation, and arousal as they visually scan the array and select appropriate responses while suppressing inappropriate ones.[10] These tasks are assigned as measures of the capacity for sustained attention, concentration, visual scanning, and rapid response activation and inhibition.[11] For others, they are measures of efficiency and speed of visual scanning,[12] or selective attention.[13,14] For yet others, they are administered primarily to assess potential hemispatial inattention and visual neglect,[15,16] or motor perseverative behaviour.[17] A recent study on the symbol cancellation test provides a measure of neglect, the organizational process, and attention.[18] Hence, the main objective of the present study was to derive normative data for the newly developed letter cancellation test.[19]

## MATERIALS AND METHODS

### Subjects

Eight hundred nineteen school students were selected in the present study in an age range between 9 and 16 years (*M* = 12.14; *SD* = 1.78 years). All of them were healthy and proficient in English. Participants were excluded from the study if they indicated that they had a history of neurological or psychiatric disturbance, and were using medication with central nervous system problems, or had a history of any learning disability. After completely describing the study to the participants, written informed consent was obtained.

### Instrument

The six letter cancellation task consisted of a test worksheet which specified the six target letters to be cancelled and had a ‘working section’ which consisted of letters of the alphabet arranged randomly in 22 rows and 14 columns. The participants were asked to cancel as many of the six target letters as possible in the specified time, *i.e*., 1 min, 30 sec. They were told to choose from two possible strategies, *i.e*., (i) doing all six letters at a time or (ii) to selective any one target letter out of the six. They were also told that they could follow a horizontal, vertical, or a random path, according to their choice.[10] The total number of cancellations and wrong cancellations were scored and the net scores were calculated by deducting wrong cancellations from the total cancellations attempted. Tests were administered by five trained assistants in the neuropsychological test laboratory.

### Data analysis

The normative procedure for net six letter cancellation scores (NSLCT) involved the fitting of multiple linear regression models adjusted for age (in years) and sex. The core assumptions of regression analysis (homoscedasticity, normal distribution of the residuals, absence of multicollinearity, and the absence of ‘influential cases’) were tested for each model. Homoscedasticity was evaluated by visual inspection of the scatter plots of the residuals on the predicted values. The normal distribution of the residuals was investigated by visual inspection of the histograms and the normal probability plots. The occurrence of multicollinearity was checked by calculating the Variance Inflation Factors (VIFs), which should not exceed 10.[20] Cook's distances were computed to identify any possible influential cases. Normative data can then be obtained by calculating the residuals for the NSLCT scores (e_i_= observed score – predicted score). The residuals are then standardized (Z_i_= e_i_/ SD [residual]). All analyses were performed by using the SPSS 10.0 version software package.

## RESULTS

Linear multiple regression models were fitted for the SLCT scores. The residuals were sufficiently normally distributed and no heteroscedasticity was observed. VIFs of the predictors in the regression models had a maximum value of 1.001, which is well below the cut-off value of 10. The outliers had virtually no effect (maximum Cook's distance 0.04). Table 1 presents the mean and standard deviation stratified by age and sex. Table 2 represents the regression models. Age and sex had a significantly positive and negative (*P* < 0.001) influence on the predicted SLCT scores.

**Table 1:** Mean and standard deviation of net six letter cancellation task scores stratified by age and sex

	Female		Male	
		
AGE (years)	n	Mean ± SD	n	Mean ± SD
9	10	16.2±6.36	17	13.06±5.03
10	71	20.73±7.1	88	16.9±6.31
11	41	23.41±6.7	84	20.62±6.21
12	49	24.35±7.96	118	22.43±8.09
13	66	31.23±9.33	74	23.7±7.63
14	31	30.81±7.53	69	26.25±8.38
15	37	34.54±9.13	43	29.93±9.87
16	9	34.67±6.18	12	29.75±12.52
Total	314	26.73±9.54	505	22.37±8.71

**Table 2:** Multiple linear regression models of the SLCT scores with age and sex as predictors

Variables	B	Std. error	t	*P* values	Standardized B	VIF	R[2]	SD (residuals)
Constant	-4.307	1.932	-2.229	0.026	-	-	-	-
Age	2.545	0.154	16.498	< 0.001	0.487	1.000	0.289	7.82
Sex	-4.250	0.563	-7.554	< 0.001	-0.223	1.000	-	-

Combining these regression models with the standard deviations of the residuals provides normative data. First, the predicted values of the scores (predicted *y*_i_) for the SLCT are calculated by inserting the coded values of the predictor variables in the regression models [Table 2]. Next, the residuals of both scores are calculated (*e*_i_ = observed *y*_i_ – predicted *y*_i_) and then standardized (*Z*_i_ = *e_i_*/ SD (residual). The SD (residual) equals 7.82 for the SLCT scores.

Multiple linear regressions provided a multiple R value of 0.538 with a corresponding R^2^ determination index of 0.29, indicating that 29% of the score variance was explained by the combination of age and sex. The model equation was: SLCT score = -4.307 + 2.545 × Age – 4.25 × Sex. This indicates that for each progressive year of age, the SLCT scores increase, on average, by 2.545 and decrease by -4.25 for each sex. These coefficients allowed us to calculate the correction scores to apply to individual subjects to consider the effects of age and sex. Table 3 provides normative SLCT data based on the regression models in Table 3, stratified by age and sex with percentile values.

**Table 3:** Net six letter cancellation tasks stratified by age and sex of raw percentile scores

		Female age in years								Male age in years							
			
	Age	9	10	11	12	13	14	15	16	9	10	11	12	13	14	15	16
	5	5	8.6	9.7	10.5	18.35	17.6	22.7	26	7	7	12	10	12	13.5	13	10
	10	5.7	11	16	13	20.7	18	24.6	26	7	9	13.00	12.90	13.50	15.00	17	11.2
	25	12	16	19	19	25	25	29.5	30	8	13	16	18	19	21	22	21
Percentiles	50	16	20	22	24	29.5	32	33	35	13	16	20.5	22	23	26	29	25
	75	23.25	27	29	31.5	38	34	38.5	38	17	22	24	27	29	32.5	35	41.75
	90	24.9	30	31	34	44	41.6	46.4	.	21	26	30	31.1	35	36	43.2	46.5
	95	.	31.8	35.6	38	47.65	43.6	54.4	.	.	28	33.75	36	36.75	39	48.8	.

### Reliability and validity

The Six Letter Cancellation test retest reliability was found (r = 0.781, *P* = 0.002).[21] This test is directly related to attention measurement. This test has been used in earlier studies in an Indian population.[22–24] Hence, this test had been validated for the present study.

## DISCUSSIONS

The results found higher scores with an increase in the age of both sexes; females had higher scores than males in the cancellation task performance. Previous studies on 50 psychiatic inpatients who had been diagnosed with substance-related disorder, schozophenia, bipolar, depressive, or anxiety disorders, showed that these patients had lower scores than normal volunteers[22] and also after coffee stimulant scores was increase.[23] To our knowledge, a prior study on the SLCT reported a general description of performance but did not provide means or standard deviations of performance on this measure for children. Moreover, the effect of demographic variables on SLCT performance had not been previously examined. However, examination of percentile ranks revealed an unstable pattern of SLCT performance across age and gender groups. Age was a stronger predictor than sex for the SLCT. This study was limited to children and uneven cell sizes across derived age and sex. Further research with larger samples is needed to clarify this relation, perhaps in an adult population. Nonetheless, these results permit quantitative evaluation of performance on the SLCT in healthy school children. As the SLCT is easy to administer in short duration of time and potentially useful in the assessment of attention, neglect, and psychomotor ability, it is hoped that these normative data will increase the use of SLCT in clinical pediatric populations.

Hence, one possible mechanism can be that the posterior parietal cortex is known to be important in normal eye movement control, visuospatial attention, and peripheral vision—all important components of reading.[25] Attention tasks that depend on parietal cortex functioning: spatial attention task,[26] perceptual grouping,[27] and visual search.[28] It is clear that many of these attention-related functions contribute to reading. Indeed, selective attention to a word or string of words requires concentrated focal attention and controlled shift of attention.

## References

[1] Laurent-Vannier A, Chevignard M, Pradat-Diehl P, Abada G, De Agostini M (2006). Assessment of unilateral spatial neglect in children using the Teddy Bear Cancellation Test. Dev Med Child Neurol.

[2] Ruff RM, Evans RW, Light RH (1986). Automatic detection vs controlled search: A paper and pencil approach. Percept Mot Skills.

[3] Halligan PW, Wilson B, Cockburn J (1990). A short screening test for visual neglect in stroke patients. Int Disabil Stud.

[4] Lowery N, Ragland JD, Gur RC, Gur RE, Moberg PJ (2004). Normative data for the symbol cancellation test in young healthy adults. Appl Neuropsychol.

[5] Lowery N, Ragland JD, Gur RC, Gur RE, Moberg PJ (2004). Normative data for the symbol cancellation test in young healthy adults. Appl Neuropsychol.

[6] Lauer CJ, Gorzewski B, Gerlinghoff M, Backmund H, Zihl J (1999). Neuropsychological assessment before and after treatment in patients with anorexia nervosa and bulimia nervosa. J Psychiatr Res.

[7] Rosselli M, Ardila A, Rosas P (1990). Neuropsychological assessment in illiterates. Brain Cogn.

[8] Kapczinski F, Curran HV, Przemioslo R, Williams R, Fluck E, Fernandes C (1996). Cognitive impairments of alcoholic cirrhotic patients: Correlation with endogenous benzodiazepine receptor ligands and increased affinity of platelet receptors. J Neurol Neurosurg Psychiatry.

[9] Richer F, Decary A, Lapierre MF, Rouleau I, Bouvier G, Saint-Hilaire JM (1993). Target detection deficits in frontal lobectomy. Brain Cogn.

[10] Sandson TA, Bachna KJ, Morin MD (2000). Right hemisphere dysfunction in ADHD: Visual hemispatial attention and clinical subtype. J Learn Disabil.

[11] Lezak MD (1995). Neuropsychological assessment.

[12] Geldmacher DS (1996). Effect of stimulus number, and target-to-distractor ratio, on the performance of random array letter-cancellation tasks. Brain Cogn.

[13] Amieva H, Lafont S, Dartigues JF, Fabrigoule C (1999). Selective attention in Alzheimer's disease: Analysis of errors in Zazzo's cancellation task. Brain Cogn.

[14] Casco C, Tressoldi PE, Dellantonio A (1998). Visual selective attention and reading efficiency are related in children. Cortex.

[15] Adair JC, Na DL, Schwarz RL, Heilman KM (1998). Analysis of primary and secondary influences on spatial neglect. Brain Cogn.

[16] Aglioti S, Smania N, Barbieri C, Corbetta M (1997). Influence of stimulus salience and attentional demands on visual search patterns in hemispatial neglect. Brain Cogn.

[17] Na DL, Adair JC, Kang Y, Chung CS, Lee KH, Heilman KM (1999). Motor perseveration behavior on a line cancellation task. Neurology.

[18] Lowery N, Ragland JD, Gur RC, Gur RE, Moberg PJ (2004). Normative data for the symbol cancellation test in young healthy adults. Appl Neuropsychol.

[19] Natu MV, Agarawal AK (1997). Testing of stimulant effects of coffee on the psychomotor performance: An exercise in clinical pharmacology. Indian J Physiol Pharmacol.

[20] Belsley DA, Kuh E, Welsch RE (1980). Regression diagnostics: Identifying the influential data and source of collinearity.

[21] Sarang PS, Telles S (2006). Comparison of psychological effect of two yoga relaxation techniques, Ph.D. Thesis.

[22] Agarwal AK, Kalra R, Natu MV, Dadhich AP, Deswa RS (2002). Psychomotor performance of psychiatric inpatients under therapy: Assessment by paper and pencil test. Hum Psycopharmacol Clin Exp.

[23] Natu MV, Agarawal AK (1997). Testing of stimulant effects of coffee on the psychomotor performance: An exercise in clinical pharmacology. Indian J Physiol Pharmacol.

[24] Sarang SP, Telles S (2007). Immediate effect of two yoga-based relaxation techniques on performance in a letter-cancellation task. Percept Mot Skills.

[25] Stein J, Walsh V (1997). To see but not to read: The magnocellular theory of dyslexia. Trends Neurosci.

[26] Brannan J, Williams MC (1987). Allocation of visual attention in good and poor readers. Percept Psychophys.

[27] Williams MC, Bologna N (1985). Perceptual grouping in good and poor readers. Percept Psychophys.

[28] Casco C, Prunetti E (1996). Visual search in good and poor readers: Effect with single and combined features targets. Percept Mot Skills.

